# Research reports from Ukraine’s frontline: provision of modular civilian medical units where facilities have been damaged, destroyed or are inaccessible

**DOI:** 10.1007/s11845-026-04447-3

**Published:** 2026-05-22

**Authors:** Gerard Bury, Shane Leahy, Miro Porochnavy, Chris Fitzpatrick, Lyudmyla Zakharchenko

**Affiliations:** 1 UCD Centre for Emergency Medical Science, University College Dublin, Dublin, Ireland; 2 One4Humanity, Carrickhill Road Upper, Poftmarnock Dublin, Ireland; 3 UCD School of Medicine, University College Dublin, Dublin, Ireland

**Keywords:** Humanitarian relief, Modern warfare, Modular medical units, Ukraine

## Abstract

**Background:**

The full-scale invasion of Ukraine by Russia in 2022 caused immediate and extensive damage to healthcare facilities, particularly those close to the frontline. A new Irish charity, driven by the business community, undertook to identify and replace facilities in key areas, using philanthropic funds.

**Methods:**

Consultation with local health authorities identified areas where facilities were destroyed, damaged or inaccessible and staff were available to continue to provide services. Purpose designed, equipped and built transportable medical modules in 20’ containers were installed. No costs were incurred by local authorities, to whom ownership and operational responsibility were transferred. Data was collected on viability, initial patient contact rates and operation into 2025.

**Results:**

19 modules were installed (eight trauma, five maternity, four primary care, two laboratory), by September2023 and most in frontline communities, at a total cost of €1.7 m. One module was unused and three had to be relocated because of nearby fighting. All active units reported patient contacts (weekly average range 4.2–80.5). Of the 19, 13 remained active in 2025, three have had to close in November 2025 due to nearby fighting and three are unknown/inactive.

**Conclusions:**

Local access to healthcare is central to the life of communities. This project developed and delivered medical modules to frontline communities in Ukraine swiftly and at reasonable cost. Many of those units remain active in 2025. The ability of a ‘pop-up’ charity to deliver ‘plug-in’, bespoke assistance may have wider application.

## Strengths and limitations of the study

A unique demonstration of the potential for rapid replacement of damaged facilities by tailormade modular units.

Provides evidence of prompt and, in some cases, extended clinical use by a wide range of healthcare services.

Suggests a role for ‘pop-up’ charitable agencies to complement established national or international humanitarian bodies.

Local feedback on clinical use of modular units is subject to ongoing conflict.

Demonstrates a specific humanitarian response to conflict, which may have limited application elsewhere.

## Introduction

The full-scale invasion of Ukraine by Russia in Feb 2022 has generated enormous disturbance to the civilian population of the country, as well as to its healthcare services. This is particularly obvious in areas where population displacement has happened and close to the frontlines where fighting may extend well into populated areas; the shifting frontlines have created even more disruption [1, 2]. One study of damage to Ukrainian healthcare facilities in the first year of the war documented 334 attacks on 267 healthcare facilities, with 37 destroyed and the rest damaged; the authors report 97 fatalities and 114 injuries in these attacks [3]. A Ukrainian Ministry of Health report ealth report indicated that in the first year of the war, 174 medical facilities were destroyed and 1,106 were damaged, mostly in the Donetsk, Luhansk, Kharkiv, Kherson and Mykolaiv districts [4]. This report indicated that almost 500 facilities were in the process of being rebuilt – however, much unmet need clearly continues to exist. This damage is in addition to significant earlier damage to healthcare facilities in many eastern districts of Ukraine caused by almost continuous fighting since 2014 [5].

The Irish medical charity One4Humanity (O4H) (RCN 20206734) was established by the business community shortly after the invasion to provide humanitarian assistance to civilians in Ukraine affected by the war and is accredited by the Irish Charities Regulator (https://www.one4humanity.world/). The charity is funded by philanthropy and businesses in Ireland and donations from businesses in other countries, particularly the United States, working in partnership with a US corporate charity Heart to Heart International (a U.S.-based 501(c)(3) non-profit organisation) (https://www.hearttoheart.org/).

Initial assessments were conducted in close collaboration with colleagues and charities working in Ukraine (particularly MedAidInternational, UK Registered Charity 1210108); these identified areas with damaged or destroyed healthcare facilities. While doctors, nurses and other healthcare professionals continued to serve their communities, many had to care for patients in severely compromised conditions with limited or no access to premises or equipment. The need for novel supports to the healthcare system was clear; in response O4H proposed the construction, purchase and installation of ‘turnkey’ modular medical units, with tailored functionality. This paper reports the content, implementation, process and initial impact of that response.

## Methods

Medical modular units were designed and developed in four types – trauma, maternity, primary care and a diagnostic laboratory, based on a standard 20’ shipping container (Appendix 1). All except one were ‘Clinic in a Can’ units supplied from the US (https://clinicinacan.com/); a final unit was a ‘Hospitainer’ supplied from The Netherlands (https://hospitainer.com/). Once agreed with local authorities, arrangements were made for shipping and location, and ownership was transferred to that authority. In some districts, more than one unit was provided, usually located separately at key areas of need.

General criteria for selection of sites included local loss of access to some or all healthcare facilities, need for additional facilities (particularly due to increased trauma cases), availability of health care professionals to provide care and (relatively) safe access for delivery of units. Monitoring of service use was carried out by agreement with a key stakeholder on each site who reported patient attendances for the initial six months after placement of the unit, together with periodic site visits by O4H staff to confirm functionality and gather feedback. ‘Active’ units were defined as those which had patient contacts during some or all of at least four weeks after delivery. ‘Inactive’ units were those which had no patient contacts or a very short duration of use. ‘Unknown’ units were those where no information could be gathered by the support team (usually due to chaotic local conditions) but where the unit may have been in clinical use. No clinical or personal data were collected at any time by or for O4H.

Data are reported here in relation to numbers, types and locations of the units provided and numbers of attendances in the period after arrival on site.

Permission has been provided by O4H and H2HI for use of these data.

## Results

**Fig. 1:**
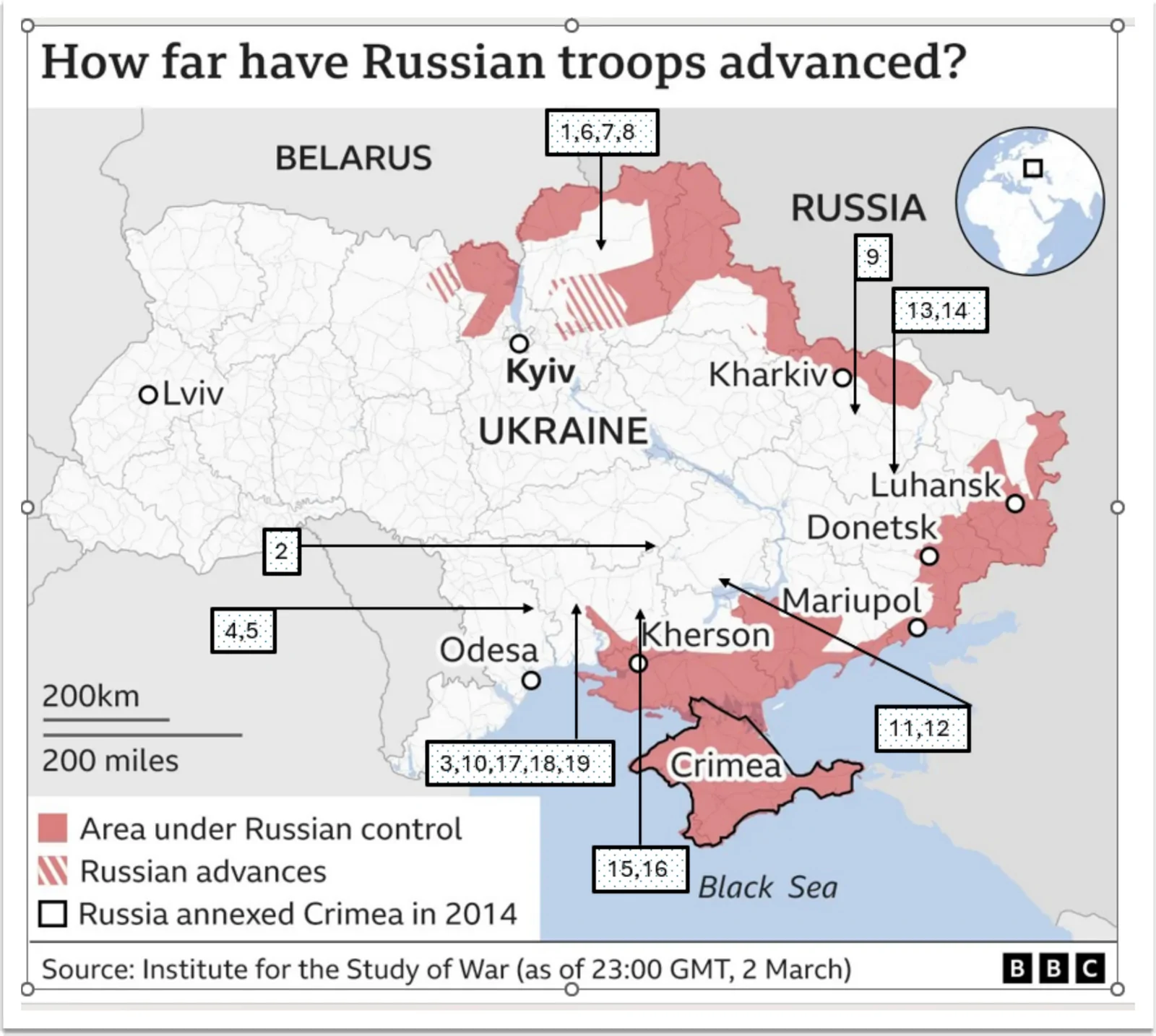
Map of Ukraine showing module sites and areas of Russian occupation, Spring 2023

In the eight-month period 2nd Jan 2023 to 1 st September 2023, 19 units were delivered to sites across the eastern part of Ukraine (Fig. 1), made up of eight trauma units, five maternity units, four primary care units and two laboratory units. The total cost was €1.7 m, with maternity units being most expensive and primary care units least expensive; communities receiving units were not asked for and did not make any financial contributions to the exercise. Most units were hosted by established primary care services within local communities, using laboratory, general assessment/primary care and trauma units. Locations vary from isolated villages to large cities.

Table 1 summarises the sites, host services, types of units and whether active when delivered in 2023 and confirmed to still be active in 2025. The map in Appendix 1 shows the geographical locations and approximate closeness to the frontlines in early 2023.

**Table 1:** Locations, host services and type of unit; activity status 2023, 2025

	City	Host service	Type	2023	2025
1	Drutske	Primary care	Laboratory	Active	Active
2	Nikopol	Primary care	Trauma	Unused	Inactive
3	Kyselivka	Primary care	Primary care	Active	Active
4	Yasnopilya	Primary care	Laboratory	Active	Active
5	Yasnopilya	Primary care	Primary care	Active	Active
6	Kolchivka	Primary care	Trauma	Active, relocated	Active
7	Novoselivka	Primary care	Primary care	Active, relocated	Unknown
8	Yahidne	Primary care	Primary care	Active	Active
9	Shevchenkove	Central hospital	Trauma	Active	Inactive
10	Mykolaiv	Maternity hospital	Maternity	Active	Active
11	Nikopol	Central hospital	Trauma	Active, relocated	Active
12	Nikopol	Primary care	Trauma/GP	Active	Inactive/safety
13	Kramatorsk	Primary care	Maternity	Active	Inactive/safety
14	Kramatorsk	Primary care	Trauma	Active	Inactive/safety
15	Kherson	Central hospital	Trauma	Active	Active
16	Kherson	Central hospital	Maternity	Active	Active
17	Mykolaiv	Central hospital	Trauma	Active	Active
18	Mykolaiv	Central hospital	Maternity	Active	Active
19	Mykolaiv	Maternity hospital	Maternity	Active	Active

One trauma unit delivered to Kramatorsk was immediately damaged by shelling but repaired and is now co-located with a new trauma centre. One unit delivered to another charity for operation was unused, due to local administrative difficulties with certification. One of the maternity units in Mykolaiv has now been co-located with a USAID funded field hospital built in 2025.

Some units are confirmed as still active in late 2025 - the primary care unit at Yahidne, for example continues to provide care for the 320 residents of the village, with 627 patient contacts reported in 2025. Yahidne was occupied by Russian forces for six months in 2022, then liberated and soon after became the host site for this unit. The unit at Drutske serves 148 residents with a weekly clinic which has seen 226 patients in 2025. A personal communication from a clinician working in the area states:

*‘The acquisition of the Mobile Clinics has greatly improved working conditions for our medical staff and has also made care more comfortable for patients, who have become accustomed to receiving medical assistance locally without the need to travel to the family outpatient clinic.*

*During doctors’ visits, electronic prescriptions for subsidized medicines under the reimbursement program are issued to patients when needed. When indicated, electronic referrals are also made for additional examinations, consultations with specialists, hospitalization, or assessment of a person’s daily functioning for the purpose of establishing a disability group.’*

A small number of units have been relocated, usually to nearby villages, following rebuilding of facilities at the original site.

Table 2 summaries activity data in the first months of operation, where known. Of the 19, 13 supplied patient contact data for at least 12 weeks (range 12–50 weeks) after the establishment of the unit. The average weekly contact rate across all units was 21.5 patients per week (range 4.2–80.5). Staff from O4H visited all units within weeks of establishment and continue to do so on a six-monthly basis where it is safe to do so. Some units are known to be well used but no data reporting arrangement could be established.

**Table 2:** Reported activity following initial placement of unit

	City	Type	2023	Period	Weeks	Patient contacts	Average/week
1	Drutske	Laboratory	Active	01/08/2023–30/11/2023	17	112	6.6
2	Nikopol	Trauma	Unused	No data provided			
3	Kyselivka	Primary care	Active	No data provided			
4	Yasnopilya	Laboratory	Active	16/01/2023–30/11/2023	45	1085	24.1
5	Yasnopilya	Primary care	Active	16/01/2023–30/11/2023			
6	Kolchivka	Trauma	Active	09/01/2023–31/07/2023	29	1332	45.9
7	Novoselivka	Primary care	Active	09/01/2023–31/07/2023	29	912	31.5
8	Yahidne	Primary care	Active	30/01/2023–16/11/2023	43	1574	36.6
9	Shevchenkove	Trauma	Active	01/09/2023–30/11/2023	12	52	4.3
10	Mykolaiv	Maternity	Active	01/05/2023–15/10/2023	24	144	6
11	Nikopol	Trauma	Active	No data provided			
12	Nikopol	Trauma/PC	Active	16/06/2023–31/12/2023	28	2254	80.5
13	Kramatorsk	Maternity	Active	01/02/2023–30/11/2023	43	616	14.3
14	Kramatorsk	Trauma	Active	No data provided			
15	Kherson	Trauma	Active	02/01/2023–31/10/2023	43	244	5.7
16	Kherson	Maternity	Active	01/02/2023–01/07/2023	12	29	2.4
17	Mykolaiv	Trauma	Active	14/01/2023–31/12/2023	50	837	16.7
18	Mykolaiv	Maternity	Active	01/02/2023–31/12/2023	44	184	4.2
19	Mykolaiv	Maternity	Active	No data provided			

As of November 2025, 13 of 19 units are known to be active, although several have suffered damage from drone and artillery strikes. Three others have been active until November 2025 but have closed because of nearby fighting.

## Discussion

This report outlines a large scale and rapid response to the destruction of established healthcare facilities close to the frontlines, after the Russian invasion in February 2022. That response was led by O4H in partnership with international business and charitable bodies using clear-cut criteria which addressed local needs; the initiative provided health care units which could be delivered on commercial lorries, were easy to install and which offered high quality facilities for use by local healthcare professionals. The overall cost of €1.7 m was accepted by donors as a reasonable cost for the outputs delivered.

Access to local healthcare is valued by all communities and the installation of these units has contributed significantly to supporting the communities served.

The willingness of installers and O4H staff to work at locations which had recently been under fire must be acknowledged – it is noteworthy that skilled volunteers from Ukraine, neighbouring countries and Ireland came forward promptly for this work.

The process was a rapid one – assessment, approval, building and installation happened within months so that many of the units were active by January 2023. Ukraine is a modern European country, with a well developed healthcare system – the installation of these modular units was essentially a ‘plug-in’ process, with seamless handover to healthcare staff who required no professional training or support to effectively provide services.

Of 19 units installed, 13 provided patient contact data for at least 12 weeks, demonstrating real utility. Full assessment of impact is limited by the absence of data from six sites (Nikopol/Trauma – never used and five others where no data was provided but phone calls indicate regular use). The absence of data usually represented areas close to the fighting and serious security concerns for all living in the area. Overall, these findings suggest an effective and sustained impact in most of the areas and services supported.

When circumstances permit a thorough review of the selection, placement and operation of the units, important lessons will be drawn, particularly in relation to risk assessment, local access and collaboration with other agencies. Contingencies such as relocation have been used for some units but other options may be identified for future use.

Avoidance of duplication and wasted effort is essential in such situations. O4H worked closely at local levels to ensure that no conflict would occur with the planned work of the Ministry of Health, other NGOs or the WHO. However, the nature of the units supplied was such that although they met an urgent need, they could not provide large scale, complex or population care and complementary plans for rebuilding nearby were therefore welcome. Some units have since been integrated into newly refurbished hospitals or clinics.

A number of additional O4H initiatives are relevant, as they arose from this initial project. O4H used contacts with the international pharmaceutical industry to access charitable donations of the (mainly) primary care oral medicines requested by hospitals and clinics across Ukraine; these supplies continue on a regular basis. The UCD Ukraine Trauma Project was supported with transport and logistics in training/equipping pre-hospital emergency care providers [6]. A large group of children with special needs in the south of the country – the ‘Key to the Future Project’—has received financial and specialist medical support and help with rebuilding their bomb damaged accommodation [7]. Finally, based on experience with modular medical units in Ukraine, a ‘Hospitainer’ fully equipped operating theatre was delivered to a partner agency in Gaza in late 2024 and has contributed a significant proportion of all surgery carried out in Gaza since then.

Our group will report on each of these projects in further publications.

O4H is a new charity with excellent international contacts, established in Ireland in mid-2022 to support Ukraine; it works closely with business agencies and charities in the US, Ukraine and Europe. Its ethos is that of the agility which characterizes good entrepreneurial skills. Is there a place for ‘pop-up’ charities like this, to complement larger, more sophisticated and longer established NGOs? O4H is run by volunteer staff (one part-time staff member in Ukraine receives a salary), drawing on volunteers with expertise in finance, logistics, law, disaster response, medicine and other disciplines as needed.

Ireland has no national, statutory relief agency (public sector healthcare staff may be allowed special leave), although many well-established charities do important work throughout the world. Internationally, the WHO Emergency Medical Teams accreditation system provides a framework to standardise medical deployments in the event of a humanitarian crisis (https://www.who.int/emergencies/partners/emergency-medical-teams/emt-global-classified-teams). Very many countries have accredited teams in one of five categories for domestic or international service; Ireland does not have such a team. The work of O4H suggests that, in a crisis, skilled and experienced volunteers are available, willing to contribute and can innovate successfully. Perhaps further exploration of this model as a complement to more formal agencies would be of value?

While progress has been made in addressing damaged healthcare facilities (by July 2025, the Ukrainian Ministry of Health reported that it had attracted around €200 m in international finance to restore its healthcare system [8]) the intervention reported here has provided effective and tailored facilities within months of the invasion and often to communities close to the frontline and in greatest need. Several years after installation, many of the units continue to be active and may continue to be useful during the phase of reconstruction.

## Appendix 1

### Container

20 ft CSC certified shipping container with an exterior surface made from 12-gauge mild steel painted with a two-part epoxy white which resists rust and reflects the sun’s heat. Insulation, interior walls, electrical and plumbing fittings interior wall, ceiling and floor coverings meet safety and working standards. Integrated heater/air conditioner. Flat panel LED lighting, 6 × universal duplex electrical outlets, external GFCI duplex outlet. Standard telephone/exterior access port access panel. Exterior water/waste water connections. Wheelchair access. 1m3 medical storage space. Fire Extinguisher 2.2 kg Class ABC rechargeable and smoke detector. Waste bins. Cabinet/sink/taps.

### Primary care medical exam room equipment

- Ritter 255 LED wall mounted Procedure Light 110/220 V 50/60 Hz
- Ritter 204 Exam table with flex drawer
- Welch Allyn GS 777 PanOptic Ophthalmoscope, MacroView Otoscope, Wall Aneroid,
- Kleenspec Specula Dispenser, Sure Temp Plus 690 Therm
- Detecto floor mounted physician scale with height rod
- Ritter 277 Airlift physician roll around stool with backrest
- Fire Extinguisher 2.2 kg Class ABC rechargeable and smoke detector
- GCX 4 I.V. bag hook M series arm wall mounted adjustable I.V. pole
- Ritter 261 Plastic Step-on 42 quart waste bin.

### Emergency/trauma unit medical equipment

- Ritter 255 LED wall mounted Procedure Light 110/220 V 50/60 Hz
- Hill-Rom 66 cm wide Trauma Gurney P8090, 3″ thick mattress, Brakes Pedal all four sides
- 02 tank holder
- GE B450 multi-para patient monitor EKG, NIBP, SP02, Pulse, Resp, Temp GCX wall arm
- Welch Allyn GS 777 PanOptic Ophthalmoscope, MacroView Otoscope, Wall Aneroid,
- Kleenspec Specula Dispenser, Sure Temp Plus 690 Therm
- Gomco Optivac G180 portable suction 110/240 V 50/60 Hz with VHM GCX wall arm
- Detecto Mechanical Eye-Level Scale, White, 180 kg Display with height rod, wheel
- Ritter 277 airlift stool with backrest
- Fire Extinguisher 2.2 kg Class ABC rechargeable and smoke detector
- GCX 4 I.V. bag hook M series arm wall mounted adjustable I.V. pole
- Drop down multipurpose wall mounted procedural tables
- GCX Wall mounted double oxygen tank holder with seismic wall channel

### Maternal/infant care unit medical equipment

- Ritter 255 LED wall mounted Procedure Light 110/220 V 50/60 Hz
- Ritter 230 Electric Procedural Table, programmable w/rotation and knee crutch
- GE 125 multi-para patient monitor EKG, NIBP, SP02, Pulse, Resp, Temp GCX wall arm
- Welch Allyn GS 777 PanOptic Ophthalmoscope, MacroView Otoscope, Wall Aneroid,
- Kleenspec Specula Dispenser, Sure Temp Plus 690 Therm
- Gomco Optivac G180 portable suction 110/240 V 50/60 Hz with VHM GCX wall arm
- Detecto Mechanical Eye-Level Scale, White, 180 kg Display with height rod, wheel
- Ritter 277 airlift stool with backrest
- GE logiq E ultrasound with cart and 4C-RS convex transducer & Sony UP D897 printer
- Fire Extinguisher 2.2 kg Class ABC rechargeable and smoke detector
- Ritter Pediatric Exam Table 640 with built-in digital scale
- Midmark M9 table top steam sterilizer 110/220 V 50/60 Hz
- undercounter refrigerator for laboratory or medical use
- GCX 4 I.V. bag hook M series arm wall mounted adjustable I.V. pole
- Drop down multipurpose wall mounted procedural tables
- GCX Wall mounted double oxygen tank holder with seismic wall channel

### Laboratory unit diagnostic equipment

- Abbott I Stat point of chemistry analyzer with printer and desk top station
- Henry Schein Microscope
- manual differential counter
- Horiba Micros 60 Hematology Analyzer automated
- Henry Schein Centrifuge bench top model 4 X 10 ml
- laboratory refrigerator
- Ritter 277 airlift stool with backrest
